# Responsiveness of the patient-specific Canadian occupational performance measure and a fixed-items activity limitations measure in patients with dupuytren disease

**DOI:** 10.1186/s41687-023-00579-7

**Published:** 2023-04-13

**Authors:** Anna Lauritzson, David Eckerdal, Isam Atroshi

**Affiliations:** 1 Department of Rehabilitation, Hässleholm Hospital, Hässleholm, Sweden; 2 Department of Orthopedics Hässleholm-Kristianstad, Hässleholm Hospital, Hässleholm, Sweden; 3 Department of Clinical Sciences - Orthopedics, Lund University, Lund, 223 62 Sweden

**Keywords:** Dupuytren, Collagenase, Hand, Occupational therapy, Outcome measure, COPM, QuickDASH, Responsiveness

## Abstract

**Background:**

Patients with Dupuytren disease experience various activity limitations. Treatment aims to reduce finger joint contractures to improve hand function and activity performance. For assessing improvement different patient-centered measures have been used. The Canadian Occupational Performance Measure (COPM) was developed as an interview-based outcome measure to detect changes over time in patients’ perception of their performance and satisfaction in self-identified activity issues. The 11-item disabilities of the arm, shoulder and hand (QuickDASH) scale consists of fixed items that ask patients to rate the difficulty in performing specific daily activities. Few studies have compared the responsiveness of these two types of patient-reported measures in Dupuytren disease.

**Patients and methods:**

We included 30 patients with Dupuytren disease enrolled in a prospective cohort study of collagenase injection. We used the COPM (score range 1–10), the QuickDASH (score range 0-100) and measurement of finger joint contracture before and 5 weeks after treatment.

**Results:**

Using the COPM the patients identified 107 activity problems (55 in self-care, 19 in productivity and 33 in leisure). The two most common activity problems were to wash self (21 patients) and to don gloves (19 patients). A clinically important improvement with 3 points or greater from baseline to 5 weeks was seen for performance in 77 activities (72%). The median COPM performance score improved from 4.4 at baseline to 9.0 at 5 weeks and the median QuickDASH score improved from 13.6 to 2.5. Responsiveness (Cohen’s d) for the COPM performance was 2.6 (95% CI 1.9–3.3) and for the QuickDASH 0.6 (95% CI 0.1–1.1).

**Conclusion:**

The COPM had about 4-fold larger responsiveness than the QuickDASH, which supports use of an individualized measure when assessing treatment effects in Dupuytren disease.

**Supplementary Information:**

The online version contains supplementary material available at 10.1186/s41687-023-00579-7.

## Introduction

In Dupuytren disease (DD) the primary and most commonly reported measure of disease severity and treatment outcome is range of motion (ROM) of affected finger joints [1, 2]. Most treatment guidelines primarily use contracture severity thresholds as an indication for treatment. This is possibly because DD usually does not cause symptoms and the effect on hand function is directly related to the joint contractures (i.e. inability to fully extend the affected fingers). However, it is generally accepted that in addition to joint ROM, measuring the impact of the disease on patients’ daily activities and quality of life is also important [2]. The most commonly used patient-reported outcome measure (PROM) in DD has been the disabilities of the arm, shoulder and hand (DASH) questionnaire or its short version, the QuickDASH [3, 4]. The activities included in the DASH were chosen to cover limitations caused by a variety of upper-extremity disorders and, thus, might not be relevant or important for the patient with DD [5, 6]. Patients with DD express a broad range of activity limitations that may not be captured with predefined items [4, 6].

In occupational therapy, the Canadian Model of Occupational Performance is a theory from which the Canadian Occupational Performance Measure (COPM) was developed as a tool to measure occupational performance. The COPM defines an occupational performance problem as “an occupation that a person wants to do, needs to do or is expected to do but cannot do, does not do or is not satisfied with the way they do” [7]. It is an individualized patient-specific outcome measure that allows patients to identify activities that they experience difficulty in performing. The COPM was developed to detect changes over time in a patients’ perception of their performance and satisfaction in self-identified activity issues. It is administered as a semi-structured interview between one patient and one interviewer. During the interview that typically takes 15 to 30 min, the patients identify up to five activities and rate their performance for each activity and how satisfied they are with their performance on a visual scale ranging from 1 (cannot perform the activity at all) to 10 (can perform the activity extremely well) [7, 8]. At follow-up reassessment, the same activities initially identified are rated regarding performance and satisfaction in a similar fashion [7, 8].

The COPM performance and satisfaction scales have been shown to have good test-retest reliability with a value above 0.80 [9] and have demonstrated convergent and divergent validity as a measure of occupational performance in the areas of self-care, productivity and leisure [10]. The COPM has been used in a wide variety of settings and as an outcome measure in research studies involving patients of various ages, disabilities and genders [11].

We have found only one published study that used the COPM in patients with DD and a variety of hand-related diagnoses [6]. To our knowledge, no previous studies have assessed the responsiveness of the COPM in patients with DD in comparison with other fixed-item measures of activity limitations.

The purpose of this study was to compare the responsiveness of the COPM and the QuickDASH in patients treated for DD.

## Patients and methods

### Study design and setting

We conducted a prospective cohort study of patients with DD undergoing treatment with collagenase injection at one orthopedic department (Hässleholm-Kristianstad Hospital) in Southern Sweden. Treatment indication was presence of a palpable cord and extension deficit of 20° or greater in the metacarpophalangeal (MCP) joint or proximal interphalangeal (PIP) joint.

### Ethics

The study was approved by the Regional Ethical Review Board. All patients provided written informed consent.

### Intervention

The treatment was given during 2 visits at the outpatient department. A hand surgeon injected collagenase into the Dupuytren cord in the palm and/or finger [12]. After an interval of 1 or 2 days the surgeon performed finger manipulation (extension) procedure under local anesthesia, aiming at best achievable reduction of joint contractures [13]. Immediately after the procedure the hand therapist equipped the patients with a static splint with fingers in maximal possible extension and gave verbal and written instructions regarding ROM exercises and oedema management. The patients were instructed to use their hand in activities and to use the splint at night for 8 weeks (in accordance with standard guidelines for collagenase treatment). The patients returned to the hand therapist 1 week after treatment for adjustment of the splint. No routine hand therapy was given.

### Measurements

At baseline (before collagenase injection) and at follow-up 5 weeks after treatment the patients completed the Swedish version of the 11-item QuickDASH questionnaire in paper form followed by the semi-structured interview for the COPM. Measurement of ROM of the fingers was then performed. All interviews and measurements were done by a single experienced hand occupational therapist (AL).

### QuickDASH

The QuickDASH provides a global assessment of upper-extremity function [14, 15]. It consists of 11 fixed items on a disability and symptom scale, 6 of which assesses performance of specific activities (open a tight or new jar, do heavy household chores, carry a shopping bag or briefcase, wash your back, use a knife to cut food, and recreational activities in which you take some force or impact through your arm, shoulder or hand such as golf, hammering and tennis). Each item has five response options (no difficulty to unable to perform) and a final score from 0 (no disability) to 100 (most severe disability) [15]. The QuickDASH has been determined to be a valid and responsive outcome measure for DD after surgery [16].

### COPM

During an individual (face to face) semi-structured interview following the Swedish translation of the 4th edition of the COPM, the patient identifies the category to which each activity issue belongs sectioned into self-care, productivity and leisure [17]. For each activity the patient answers the question “How do you rate your performance of this activity?” on a visual rating scale card, with responses ranging from 1 (cannot perform the activity at all) to 10 (can perform the activity extremely well) (supplemental Figure). Similarly, the patient rates satisfaction with the performance of each activity by answering the question: “How satisfied are you with the way you are able to perform this activity now?” from 1 (dissatisfied) to 10 (extremely satisfied) [7]. The examiner records the patient’s responses, on a COPM score sheet, for both performance and satisfaction for each activity before proceeding to the next activity. At follow-up 5 weeks after treatment patients were informed verbally about which activities they had identified at baseline, and were asked to score performance and satisfaction for the same activities, without the baseline score being revealed to the patient [17].

### Joint contracture

Active ROM was measured for each joint in the affected finger with a finger goniometer (Smith and Nephew Rolyan Inc, Germantown, WI, USA), following the guidelines proposed by the American Academy of Orthopaedic Surgeons [18]. The goniometer was placed dorsally over the joint to be measured [19]. To prevent underestimation of the total extension deficit, hyperextension at individual joint level was converted to 0°.

### Statistical analysis

For each activity the baseline COPM score was subtracted from the 5-week follow-up score, for both performance and satisfaction. A change of 2 or more points on the COPM scale between the first and second rating has been considered clinically important [20]. However, later research has suggested that 3 points is a more certain clinical change [21].

We calculated improved score based on both ≥ 3 points and ≥ 2 points. We also calculated median scores and quartiles at baseline and follow-up for the COPM and the QuickDASH and analyzed the change with the Wilcoxon test. We calculated mean total active extension deficit (TAED) defined as the sum of AED for the MCP and PIP joints of the treated fingers. We also analyzed the correlations between TAED and COPM and QuickDASH scores with the Spearman correlation coefficient. As a measure of responsiveness, we used Cohen’s d: values of 0.2 indicate small, 0.5 moderate and ≥ 0.8 large clinical change [22]. Because the cause of activity limitations in DD is almost exclusively finger joint contractures (DD in itself is usually asymptomatic) it would be reasonable to assume that if the contractures resolve, the activity issues caused by the contractures should improve. We therefore considered TAED of the treated finger as the criterion for comparison. The analyses were performed with STATA v 16.0 (Stata Corporation, College Station, TX). A p-value < 0.05 was considered to indicate statistical significance.

## Results

### Patients

The study included 32 consecutive patients treated with collagenase injection. One patient had two fingers in the same hand treated with a 5-week interval between treatments. Because the patient reported problems with different activities before each treatment both fingers were included in the study. One patient residing outside the study region could not return for follow-up on account of the long travel distance. Thus, 5-week follow-up was conducted on 30 patients (21 men and 9 women; 31 treated fingers), with a mean age of 67 years (Table 1). Of the treated fingers, baseline contracture was present in 30 MCP and 18 PIP joints.

**Table 1:** Patient characteristics

	All	Men	Women
No. of patients (n treated fingers) ^a^	30 (31)	21 (22)	9 (9)
Age, median (range) y	67 (55–79)	67 (55–79)	67 (62–77)
Retired, n (%)	25 (83)	17 (81)	8 (89)
Dominant hand treated, n (%)	21 (70)	14 (67)	7 (78)
Treated finger, n (%)			
Small	19 (61)	13 (59)	6 (67)
Ring	12 (39)	9 (41)	3 (33)

^a^ One patient had 2 fingers in same hand treated on 2 separate occasions

### COPM

At baseline the patients identified 107 activities as problematic and scored them regarding performance and satisfaction. Each patient identified between 1 and 5 activities as problematic: 1 activity (n = 1), 2 activities (n = 11), 3 activities (n = 3), 4 activities (n = 5) and 5 activities (n = 11). The two most common activity problems were to wash self (n = 21) and to don gloves (n = 19) (Table 2). The third most common problematic activity was shaking hands (n = 8). Other activities identified as problems were to use a computer keyboard (n = 7), put hand in a pocket, grasp a container and wipe off a table (n = 4), grasp a steering wheel, apply lotion, button, grab items and wash or polish a car (n = 2). In addition, 26 activities were unique problems for individual patients.

**Table 2:** All activities identified by the patients in each of the three areas of the Canadian Occupational Performance Measure (COPM).

Area/Activity	Performance				Satisfaction		
	n	Improved 3 points	Improved 2 points	Unchanged / Worse*	Improved 3 points	Improved 2 points	Unchanged / Worse*
**Self-care**							
Wash-self	21	15	3	3	14	4	3
Don gloves	15	12	2	1	11	2	2
Put hand in pocket	4	4	0	0	3	1	0
Grasp container i.e. glass, mug	4	3	0	1	3	0	1
Hold steering wheel	2	1	0	1	1	0	1
Put on lotion	2	2	0	0	1	1	0
Button	2	1	0	1	1	0	1
Grasp items	2	0	1	1	1	0	1
Hand break on motorcycle	1	1	0	0	1	0	0
Wash hair	1	1	0	0	1	0	0
Let go of door handle	1	1	0	0	1	0	0
**Productivity**							
Wipe table	5	4	1	0	4	0	1
Use computer keyboard	4	2	1	1	2	0	2
Open jar	2	1	0	1	0	1	1
Don work glove	2	2	0	0	2	0	0
Maneuver hand in limited space	1	0	0	1	1	0	0
Bake	1	1	0	0	1	0	0
Clean window	1	0	0	1	0	0	0/1
Write	1	1	0	0	1	0	0
Give massage	1	0	0	1	0	0	1
Chop onion	1	0	0	1	0	0	1
**Leisure**							
Shake hands	8	6	2	0	6	2	0
Use computer keyboard	3	2	1	0	1	1	1
Wash or polish car	2	2	0	0	1	0	1
Don work gloves	2	1	0	0/1	1	0	0/1
Applaud	1	1	0	0	1	0	0
Play guitar	1	1	0	0	1	0	0
Repair car	2	0	2	0	1	1	0
Caress	1	1	0	0	1	0	0
Restore house	1	1	0	0	0	1	0
Sculpture	1	1	0	0	1	0	0
Hold a book	1	1	0	0	1	0	0
Play golf	1	1	0	0	1	0	0
Play saxophone	1	1	0	0	1	0	0
Push-ups	1	1	0	0	1	0	0
Let go of door handle	1	1	0	0	1	0	0
Walk the dog	1	0	1	0	0	0	0/1
Swing grandchildren on a swing	1	1	0	0	1	0	0
Dig	1	1	0	0	1	0	0
Saw	1	1	0	0	1	0	0
Handle a rifle	1	1	0	0	1	0	0
Tie a fly	1	0	1	0	0	1	0

* Unchanged is score change of + 1, 0, -1 point and worsened is score change of -2 or more points

Of all 107 identified activity problems 55 (51%) were in self-care, 19 (18%) in productivity and 33 (31%) in leisure. The activity to don gloves was a problem in all three areas, with knitted or inelastic leather gloves mentioned as the problem in self-care and work gloves in productivity and leisure. Computer keyboard use was identified as a problem in both productivity and leisure, depending on whether the computer was used for work or at home.

Between baseline and 5 weeks the COPM scores improved by ≥ 3 points for performance in 77 activities (72%) and for satisfaction in 72 activities (67%) (Table 3). Improvement by ≥ 2 points occurred for performance in 92 activities (86%) and for satisfaction in 87 (81%). An unchanged COPM score (score change of + 1, 0, -1 point) for performance was found in 14 activities (13%) and for satisfaction in 17 activities (16%). A worsened score (score change of ≥-2) for performance was found in 1 activity (1%) and for satisfaction in 3 activities (3%). Of the 30 patients, 23 scored improved performance (by ≥ 2 points) in all their identified activities, 6 patients scored improved performance in 14 activities and unchanged performance in 9 activities, 1 patient scored unchanged performance in all activities, and 1 patient scored improved performance in 2 activities and worsening in 1 activity.

The mean change in COPM performance score from baseline to 5 weeks was − 4.0 (95% CI -4.6 - -3.5).

**Table 3:** Change in the COPM performance (P) and satisfaction (S) scores between baseline and 5 weeks in the identified activities within each of the three areas

	All activities (n = 107)		Self-care (n = 55)		Productivity (n = 19)		Leisure (n = 33)	
Points*	P n (%)	S n (%)	P n (%)	S n (%)	P n (%)	S n (%)	P n (%)	S n (%)
9	4 (4)	13 (12)	3 (5)	10 (18)		2 (11)	1 (3)	1 (3)
8	4 (4)	8 (8)	1 (2)	3 (5)	1 (5)	1 (5)	2 (6)	4 (12)
7	14 (13)	13 (12)	9 (16)	9 (16)	2 (11)	2 (11)	3 (9)	2 (6)
6	9 (8)	9 (8)	6 (11)	5 (9)			3 (9)	4 (12)
5	18 (17)	13 (12)	12 (22)	5 (9)	1 (5)	3 (16)	5 (15)	5 (15)
4	20 (19)	9(8)	6 (11)	4 (7)	5 (26)	1 (5)	9 (27)	4 (12)
3	8 (8)	7 (7)	4 (7)	2 (4)	2 (11)	2 (11)	2 (6)	3 (9)
2	15 (14)	15 (14)	6 (11)	8 (15)	2 (11)	1 (5)	7 (21)	6 (18)
1	3 (3)	6(6)	2 (4)	3 (5)	1 (5)	2 (11)		1 (3)
0	10 (9)	10 (9)	6 (11)	5 (9)	4 (21)	4 (21)		1 (3)
-1	1 (1)	1(1)		1 (2)	1 (5)			
-2	1 (1)	1 (1)					1 (3)	1 (3)
-3		1 (1)				1 (5)		
-4		1 (1)						1 (3)

*Points on a scale from 1 (worst) to 10 (best), score change is follow-up score minus baseline score

### QuickDASH

The median QuickDASH score improved from 13.6 at baseline to 2.5 at 5-week follow-up. (Table 4). The mean change in QuickDASH score was 8.4 (95% CI 4.3–12.4).

**Table 4:** Active extension deficit (degrees) in treated finger joints, and scores for the QuickDASH and COPM

Measure^*^	Baseline median (quartiles)	5 weeks median (quartiles)	Change^†^ median (quartiles)
TAED	85 (60, 115)	20 (0, 40)	-65 (-85, -50)
QuickDASH	13.6 (2.3, 20)	2.5 (0, 9.1)	-9.1 (-11.4, -2.3)
COPM-P	4.4 (3.4, 5.5)	9.0 (8.0, 10.0)	4.0 (3.0, 5.2)
COMP-S	3.6 (2.6, 5.5)	9.2 (8.0, 10.0)	4.5 (2.4, 5.6)

^*^QuickDASH score range from 0 (best) to 100 (worst), COPM-P and COPMS score range from

1 (worst) to 10 (best)

^†^P > 0.001 for all analyses

COPM, Canadian Occupational Performance Measure (P = Performance, S = Satisfaction); QuickDASH, 11-item Disabilities of the Arm, Shoulder and Hand; TAED, total active extension deficit (metacarpophalangeal joint + proximal interphalangeal joint)

### Joint contracture

Active extension of the treated joints improved significantly (Table 4). For the MCP joints mean AED improvement (degrees) was 52 (95% CI 42–61) and for the PIP joints was 15 (95% CI 4–27); mean change in TAED was 67 (95% CI 58–75).

A moderate correlation was found between TAED and COPM performance score at baseline (r= -0.45, p = 0.011) and 5 weeks (r = 0.42, p = 0.017). A lower correlation was found between TAED and QuickDASH score at baseline (r = 0.37, p = 0.047) and 5 weeks (r = 0.32, p = 0.089).

### Responsiveness

Cohen’s d for TAED was 2.1 (95% CI 1.5–2.7), for QuickDASH 0.6 (95% CI 0.1–1.1) and for COPM performance 2.6 (95% CI 1.9–3.3).

## Discussion

This study showed that the COPM, a patient-specific measure of activity limitations, had a much higher responsiveness (about 4-fold as measured with Cohen’s d) than the fixed-items QuickDASH. The higher responsiveness may be related to the fact that patients with DD experience limitations in different types of activities. A systematic review of patient-reported outcome measures in patients with DD found that the reported effect size for the QuickDASH was 0.54 to 0.64, which is similar to that found in our study [23].

In our study, 30 patients identified 107 different activities as problematic, with a mean of 3.6 per patient. In 2 previous studies patients with DD identified a mean of 2.9 out of 5 activities, [24] and 2.5 out of 3 activities per patient as problematic [4]. All patients in our study identified at least 1 activity as problematic at baseline suggesting that the reason for the patients to seek treatment was related to activity issues and not as much for aesthetic reasons as suggested in a previous study [25]. Of all the activities identified by the patients, 26 were unique for that individual. This is not surprising as a patient-specific method was used and the findings are consistent with a variety of activity problems experienced by patients with DD [5, 24].

The activities rated most frequently as problematic were within self-care, mainly to wash self, don gloves and put hand in pocket. These findings are similar to those of previous studies in which difficulty washing self was also the most common problem specified by patients in a study from the UK [4] and to don gloves and put hand in pocket identified as specific problems in a study from the Netherlands [24]. Donning gloves in our study was identified as an activity issue in all three sections of the COPM (finger-knitted or leather gloves within self-care and work gloves within productivity and leisure). In the study from the UK 23% of patients experienced difficulties putting on gloves and 8% had difficulties putting hands in pocket [4]. Difficulty using computer keyboard was identified both in the areas of productivity and leisure; similarly, this activity was experienced as very problematic by patients in the studies from the UK and the Netherlands [4, 24].

The COPM showed that the majority (72%) of patients with DD rated their performance of the activities they had identified before treatment as improved (score increase by 3 or more points) at 5 weeks after treatment. In addition, satisfaction with performance of these activities had improved in 67%. A previous study that used the COPM found higher satisfaction scores than performance scores in patients with DD [6]. This may suggest that patients assess their hand function and their satisfaction with this function separately, a distinction not possible with QuickDASH.

In our study only two fixed-item activities (to open a jar and to play golf) in the QuickDASH were identified as an issue using COPM. Although the mean baseline score in our study was not as high as in some other conditions of the hand, [15] the QuickDASH still showed a statistically significant and clinically moderate improvement after treatment.

In our study, the joint contractures in the treated fingers had decreased significantly at 5 weeks after collagenase treatment, with a mean improvement of 67° in TAED (52° in MCP joints and 15° in PIP joints). The mean TAED at 5 weeks was 23°, which is comparable to fasciectomy, after which a TAED of 24° was reported at 12 weeks after surgery, as well as a previous study from our center where a total extension deficit of 21° was reported at 5 weeks after extension [5, 26]. Thus, the patients in this study appear to be representative of patients with DD requiring treatment.

Considering that COPM requires in-person interview it may not be practical for use in population studies. However, it has been suggested that the unique information provided by COPM justifies the time and resources needed for its administration [27]. The results of the present study with a large difference in responsiveness compared to a fixed-item PROM further shows the benefits of the COPM. With its larger responsiveness, use of the COPM reduces the sample size needed in clinical trials that aim to compare the efficacy of different treatment methods.

Our study had several limitations. We did not take into consideration possible presence of contracture in other fingers in the treated hand or in the non-treated hand. Hence, we could not determine if their treated joints caused their remaining activity problems after treatment.

The use of the QuickDASH for comparison with the COPM is another limitation as it is a region-specific PROM and not disease-specific, thus possibly introducing bias due to the potential influence of other injuries or non-DD related disabilities in the upper extremities. However, the QuickDASH was chosen because it is the most commonly used PROM in Dupuytren research. Also in a recent systematic review the effect size for the QuickDASH (2 studies with 594 subjects) was 0.54 and 0.64, respectively, and that for the Unité Rheumatologique des Affections de la Main (URAM) scale ranged from 0.56 to 0.96 (3 studies with 199 subjects) [23]. Moreover, in another recent study, disease-specific PROMS such as the Southampton Dupuytren’s scoring scheme (SDSS) and the URAM did not perform better than hand-specific PROMs, with effect sizes (calculated as mean change in score / baseline SD) of approximately 1.0 for both URAM and SDSS, values substantially lower than those found for COPM in the present study [28]. Finally, the relevance of the URAM in Dupuytren research has been questioned [4].

Another factor that may have influenced the results is that the patients might have been made more aware of their activity performance and satisfaction after the first COPM interview, which may have caused lower scoring after treatment. Patients may give answers that they think are expected from them [29], or may bring up activity problems that are not related to the disease being assessed. On the same subject, the interview was conducted by the treating occupational hand therapist and not a neutral party, which may influence the responses.

Since patients rate the same activities after treatment as they did at baseline, it is possible that the activities the patient may have difficulty in performing after treatment may not be the same activities they listed before treatment.

A possible limitation is that all patients were advised to use night splint and given a written exercise program which could have affected the results. Furthermore, we did not record to what extent the splint was used or the instructions followed. Also, since the mean age of the patients was 67 years and most of them were retired, it is possible that the results would be different in a younger cohort. However, because the prevalence of DD increases with age, our data provide information about activity limitations in an age group representative of the disease [30]. Although the sample size was small, the difference in responsiveness between the COPM performance and the QuickDASH as measured with Cohen’s d was very large, even considering the 95% confidence intervals.

## Conclusion

The COPM had 4-fold larger responsiveness than the QuickDASH. This supports the use of an individualized measure when assessing treatment effects in DD.

### Electronic supplementary material

Below is the link to the electronic supplementary material.

Supplementary Material 1

## Data Availability

The data are available upon request to the corresponding author.

## List of abbreviations

DD: Dupuytren disease
ROM: Range of Motion
PROM: Patient-reported outcome measure
DASH: Disabilities of the arm, shoulder and hand
COPM: Canadian Occupational Performance Measure
MCP: Metacarpophalangeal
PIP: Proximal interphalangeal
AED: Active extension deficit
TAED: Total active extension deficit
