# Correction: Responsiveness of the patient-specific Canadian occupational performance measure and a fixed-items activity limitations measure in patients with dupuytren disease

**DOI:** 10.1186/s41687-024-00768-y

**Published:** 2024-08-06

**Authors:** Anna Lauritzson, David Eckerdal, Isam Atroshi

**Affiliations:** 1 Department of Rehabilitation, Hässleholm Hospital, Hässleholm, Sweden; 2 Department of Orthopedics Hässleholm-Kristianstad, Hässleholm Hospital, Hässleholm, Sweden; 3 Department of Clinical Sciences - Orthopedics, Lund University, Lund, 223 62 Sweden

**Correction to: Journal of Patient-Reported Outcomes 7**,** 38 (2023)**

https://doi.org/10.1186/s41687-023-00579-7

Following publication of the original article it was reported that there was a recurring typographical error where ‘6-fold’ was written instead of ‘4-fold’ in three instances, as listed below with the corrected text in bold:

In the Abstract, the Conclusion section should read: The COPM had about **4-fold** larger responsiveness than the QuickDASH, which supports use of an individualized measure when assessing treatment effects in Dupuytren disease.

The first sentence of the Discussion section should read: This study showed that the COPM, a patient-specific measure of activity limitations, had a much higher responsiveness (about **4-fold** as measured with Cohen’s d) than the fixed-items QuickDASH.

The article Conclusion should read: The COPM had **4-fold** larger responsiveness than the QuickDASH. This supports the use of an individualized measure when assessing treatment effects in DD.

The original article has been updated.

