# Supplementary material for: Responsiveness of the patient-specific Canadian occupational performance measure and a fixed-items activity limitations measure in patients with dupuytren disease
Source: J Patient Rep Outcomes. 2023 Apr 13;7:38. doi: 10.1186/s41687-023-00579-7 (PMC10102265; doi:10.1186/s41687-023-00579-7)
Supplement: Supplementary file 1 — Supplementary Material 1 [file 41687_2023_579_MOESM1_ESM.docx]

| **Utförande** | | | | | | | | | | |
| --- | --- | --- | --- | --- | --- | --- | --- | --- | --- | --- |
|  |  | |  |  |  |  |  |  |  |  |
| 1 | 2 | | 3 | 4 | 5 | 6 | 7 | 8 | 9 | 10 |
|  | | | | | | | | | | |
| Kan inte utföra den alls | |  | | | | | | | Kan utföra den extremt bra | |
| The Swedish COPM rating scale card used in the study to rate the Performance scale.  Translations from the original COPM English version:  Utförande = Performance  Kan inte utföra den alls = Not able to do it at all  Kan utföra den extremt bra = Able to do it extremely well. | | | | | | | | | | |

| **Tillfredställelse** | | | | | | | | | | | |
| --- | --- | --- | --- | --- | --- | --- | --- | --- | --- | --- | --- |
|  |  | |  |  |  |  |  |  |  | |  |
| 1 | 2 | | 3 | 4 | 5 | 6 | 7 | 8 | 9 | | 10 |
|  | | | | | | | | | | | |
| Inte nöjd alls | |  | | | | | | | | Extremt nöjd | |
| The Swedish COPM rating scale card used in the study to rate the Satisfaction scale.  Translations from the original COPM English version:  Tillfredställelse = Satisfaction  Inte nöjd alls = Not satisfied at all  Extremt nöjd = Extremely satisfied | | | | | | | | | | | |
